# Automated preparation of clinical grade [^68^Ga]Ga-DOTA-CP04, a cholecystokinin-2 receptor agonist, using iPHASE MultiSyn synthesis platform

**DOI:** 10.1186/s41181-019-0067-2

**Published:** 2019-08-23

**Authors:** Mohammad B. Haskali, Peter D. Roselt, David Binns, Amit Hetsron, Stan Poniger, Craig A. Hutton, Rodney J. Hicks

**Affiliations:** 1 The Centre for Molecular Imaging and Translational Research Laboratory, The Peter MacCallum Cancer Centre, Melbourne, Victoria Australia; 2 Sir Peter MacCallum Department of Oncology, The University of Melbourne, Victoria, 3010 Australia; 3 iPHASE Technologies Pty. Ltd., Melbourne, Australia; 4 School of Chemistry, The University of Melbourne, Victoria, 3010 Australia; 5 Bio21 Molecular Science and Biotechnology Institute, The University of Melbourne, Victoria, 3010 Australia

**Keywords:** [^68^Ga]Ga-DOTA-CP04, Neuroendocrine tumor, Somatostatin receptor, CCK-2, positron emission tomography

## Abstract

**Background:**

Gallium-68 ([^68^Ga]Ga) labelled radiopharmaceuticals have become a valuable tool in clinical practice using Positron Emission Tomography (PET). These agents are typically produced on-site owing to the short half-life of [^68^Ga]Ga (68 min), which hinders distant transportation and often cannot comply with Good Manufacturing Practice (GMP) in hospital environments due to limited resources or infrastructure constraints. Moreover, full blown GMP production of radiopharmaceuticals under development can be prohibitively expensive. [^68^Ga]Ga-DOTA-CP04 is a promising peptide for imaging neuroendocrine tumors overexpressing the cholecyctokinin-2 receptor. Automation is an integral process in ensuring the radiopharmaceuticals produced under non-GMP conditions are of a uniform quality for routine clinical use. Herein, we describe the development of an automation platform, the iPHASE MultiSyn radiosynthesizer, to produce ^68^Ga-labelled DOTA-CP04 for routine clinical provision.

**Results:**

The use of the MultiSyn module for ^68^Ga-labelling of DOTA-CP04 was investigated. [^68^Ga]Ga-DOTA-CP04, was reproducibly prepared in high (> 70%) decay-corrected yields. [^68^Ga]Ga-DOTA-CP04 passed all predetermined acceptance criteria for human injection.

**Conclusions:**

[^68^Ga]Ga-DOTA-CP04 was produced effectively using the MultiSyn module in a consistent and reproducible manner suitable for human injection.

**Electronic supplementary material:**

The online version of this article (10.1186/s41181-019-0067-2) contains supplementary material, which is available to authorized users.

## Background

^68^Ga-labelled radiopharmaceuticals are increasingly being used for positron emission tomography (PET) imaging in oncology due to a number of ligands that have demonstrated high-affinity and specific target localization with rapid background clearance (Brandt et al., 2018). Furthermore, the utility of [^68^Ga]Ga circumvents the need for an on-site cyclotron since it is produced from a ^68^Ge/^68^Ga generator (Banerjee & Pomper, 2013). This makes ^68^Ga-labelled radiopharmaceuticals highly accessible, even in facilities without extensive radiopharmaceutical infrastructure but may, as a consequence, give rise to variability in radiopharmaceutical quality. Therefore, it is necessary to develop methods that ensure the radiopharmaceuticals produced exhibit uniform quality (Kristensen, 1979). This is particularly important for multicenter clinical trials.

Some of the available ^68^Ga-radiopharmaceuticals target rare diseases and are therefore unattractive for commercial radiopharmacies. Consequently, their availability is often restricted to hospital environments that are constrained in their ability to operate under comprehensive GMP guidelines due to cost, staffing and infrastructure constraints. However, the quality of these radiopharmaceuticals must not be compromised for human studies. Furthermore, the short half-life of most radiopharmaceuticals, including those radiolabeled with [^68^Ga]Ga, adds extra constraints to the overall production and quality control processes. It is therefore essential to produce radiopharmaceuticals in highly organized environments using rapid and well-established procedures (Kristensen, 1979). In this arena, automating the production of radiopharmaceuticals is a pivotal development that ensures the uniform quality of the end-product while minimizing the radiation burden on the operating radiochemist (Elsinga et al., 2010).

As an example of this process of automation of a “niche” ^68^Ga-labelled radiotracer, we describe the automation of synthesis of [^68^Ga]Ga-DOTA-CP04, which is a promising radiopharmaceutical under study for the imaging of tumors overexpressing cholecystokinin-2 receptors (CCK-2R). PET imaging of CCK-2 overexpression has presented clinical utility in diagnosing neuroendocrine tumors (NETs) with low somatostatin-2 receptor expression, particularly for the staging of medullary thyroid carcinoma. CP04 is a modified and potent 13-amino acid peptide derived from the gastrin hormone and retains the C-terminal message sequence Trp-Met-Asp-Phe-NH_2_ responsible for binding to the receptor. In CP04, the N-terminal sequence has been modified to replace the 5 N-terminal L-glutamic acid residues present in the native gastrin hormone with 6 D-glutamic acid residues. The modification from L to D glutamic acid residues added considerable stability to the peptide and resulted in minimal kidney uptake and retention of the radiopharmaceutical (Laverman et al., 2011). Extensive reports have described the [^111^In] Inlabelling of CP04 (and its analogues) and its stabilization for gastrin scintigraphy of patients (Laverman et al., 2011; Breeman et al., 2008; Fröberg et al., 2009; Aloj et al., 2011). However, no controlled and/or extensive production conditions have been reported for producing [^68^Ga]Ga-DOTA-CP04 for clinical positron emission tomography (PET). Herein, we describe the first-reported, fully-automated production of [^68^Ga]Ga-DOTA-CP04 injection using a radiosynthesis module (iPHASE MultiSyn, (Additional file 1: Figure S1)).

## Methods

Chemicals were of European Pharmacopeia grade where applicable. [^68^Ga]Ga was eluted from an ITG ^68^Ge/^68^Ga generator (ITG, Munich, Germany) using 4 ml of 0.05 M HCl. Production was performed on an iPHASE MultiSyn module with hardware cassettes and ancillaries kit (iPHASE, Melbourne, Australia) and all starting reagents being GMP-certified (Huayi Isotopes (Suzhou, China). DOTA-CP04 was prepared in-house using Fmoc-solid phase peptide synthesis protocols and was rigorously purified and characterized (see Figure S4 and S5 of the Additional file 1 for LC-MS and MS/MS characterization data). CRC-15PET dose calibrator (Capintec) was calibrated using Cs-137 and Co-57 sources (Isotope Products Laboratories) and used for radioactivity measurements. Gas Chromatography (GC) analysis was performed on a Shimadzu GC-17A instrument coupled with a AOC-20i auto injector. Radio-TLC analyses were performed using a Raytest Rita-Star TLC scanner using Varian iTLC-SG silica gel impregnated glass microfiber chromatography paper. Radio-HPLC analyses were performed using a Shimadzu HPLC (SCL-10AVP system controller, SIL-10ADVP auto injector, LC-10ATVP solvent delivery unit, CV-10AL control valve, DGU-14A degasser, and SPD-10AVPV detector, Kyoto, Japan) coupled to a scintillation detector (Ortec 276 Photomultiplier Base with Preamplifier, Ortec 925-SCINT ACE mate Preamplifier, Amplifier, BIAS supply and SCA, and a Bicron 1 M 11/2 Photomultiplier Tube). Sterility and endotoxin testing were outsourced to Eurofins Scientific Testing laboratory company.

### [^68^Ga]Ga-DOTA-CP04

**Fig. 1:**
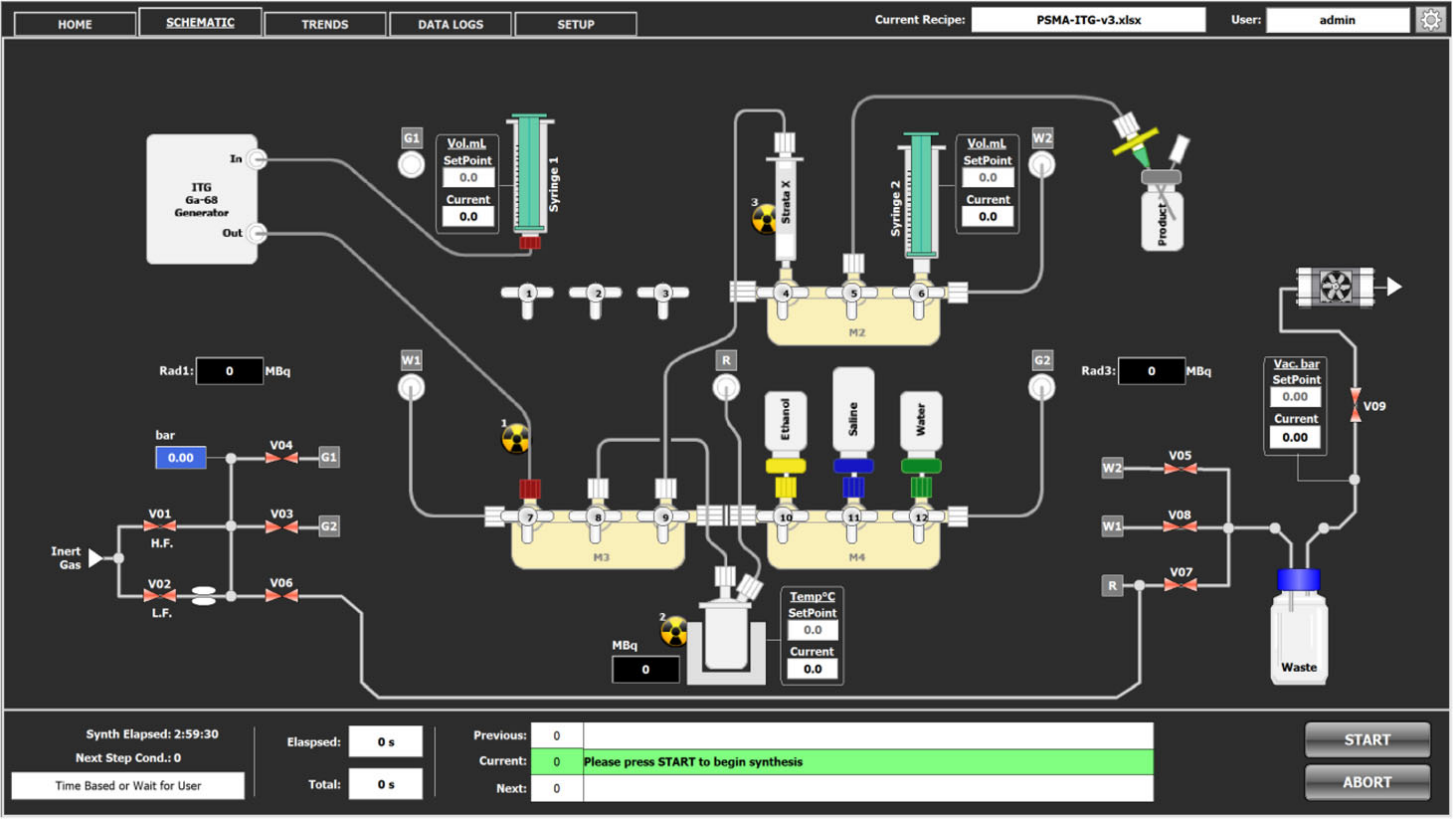
Schematic of the MultiSyn module for production of Ga-68 labelled radiopharmaceuticals

Sterile production cassettes were mounted on the MultiSyn manifolds (see physical representation of the module in Additional file 1: Figure S1) and automatically tested for leaks. A 0.2 μm Millex-FG Filter was installed at the gas inlet (port G2, see Fig. 1 for illustration) thereby ensuring sterile filtration of incoming nitrogen. Product transfer lines were rinsed with sterile 70% ethanol prior to any production commencement and dried with sterile filtered HP nitrogen. A mixture of DOTA-CP04 precursor (30 μg) in 0.5 M sodium acetate solution (800 μL), ethanol (200 μL), 0.05 M sodium ascorbate (200 μL), 0.05 M 2,5-dihydroxybenzoic acid sodium salt (200 μL) and 10 mg/ml methionine (100 μL) was prepared immediately before production in a laminar flow hood. The mixture was transferred into the reactor of the MultiSyn cassette and the automated process started. The ITG ^68^Ge/^68^Ga generator was eluted directly into the MultiSyn synthesizer reactor using 0.05 M HCl (4 ml). Radiolabeling was performed at 95 °C for 480 s (pH of reaction mixture is 4.5). At the completion of labelling, the reaction mixture was diluted with water (5 ml) and trapped on a Strata-X SPE cartridge. The trapped product was rinsed with water (5 ml), eluted with ethanol (~ 0.5 ml) and diluted with saline (2 ml). [^68^Ga]Ga-DOTA-CP04 was delivered into a sterile vial through a 0.22 μm Cathivex-GV 25 mm PVDF sterile filter. A further portion of saline (7 ml) was used to rinse the delivery lines into the sterile vial to afford the final product in ≤10% ethanol in saline.

### HPLC radiochemical purity and identity assessment

Radio-HPLC analysis of [^68^Ga]Ga-DOTA-CP04 for injection and its non-radioactive reference standard was performed using the following chromatographic conditions: Kinetex XB C18 column (5 μm, 100 Å, 250 × 4.60 mm) eluted at 1 ml/min with a gradient of MeCN: 0.05% (v/v) TFA, starting at 25% MeCN for 1 min, increased to 90% B over 10 min and maintained at 90% MeCN for 5 min. The radiochemical identity was confirmed by matching retention time (and co-mobility) of [^68^Ga]Ga-DOTA-CP04 and its non-radioactive reference standard. The radiochemical purity is identified by rigorous integration of all observed radioactive peaks and comparison of their relative % area.

### TLC radiochemical purity assessment

Radio-TLC was performed using ITLC-SG strips (2 cm width × 10 cm length). A small drop of [^68^Ga]Ga-DOTA-CP04 for injection is placed on an ITLC-SG strip 2 cm above the base line. The strip is then placed in a chamber containing aqueous 1 M NH_4_OAc in methanol (1:1) as mobile phase. The mobile phase is allowed to migrate to approximately 1 cm below the top end of the strip. It is then removed and placed into a Raytest TLC reader to determine % area of radioactivity at the origin (non-migrated, representing free [^68^Ga]Ga) and solvent front (representing peptide related labelled material).

### GC analysis of ethanol content

GC analysis was performed on [^68^Ga]Ga-DOTA-CP04 for injection by diluting the samples 10 fold in water. Area % attained for ethanol in the diluted samples was multiplied by 10 and related to a calibration curve constructed for 10–0.01% ethanol in water to determine total ethanol content in the samples for injection.

## Results

The automated production of [^68^Ga]Ga-DOTA-CP04 was completed in 22 min from the time the ITG generator was eluted (Additional file 1: Figure S6 displays a representative MultiSyn sequence used for the production of [^68^Ga]Ga-DOTA-CP04). [^68^Ga]Ga-DOTA-CP04 was prepared reproducibly in 74.8% ± 3.4% (*n* = 10) decay-corrected yield. Initial [^68^Ga]Ga activity was in the range of 370–1887 MBq affording 216–1063 MBq of [^68^Ga]Ga-DOTA-CP04 product. The initial level of activity did not influence the observed radiochemical purity or yield. The final product was formulated in saline containing ≤10% ethanol and was found to be stable for at least 2 h in this formulation. The stability of the radiopharmaceutical in this formulation was verified using HPLC, TLC, pH and by appearance.

**Fig. 2:**
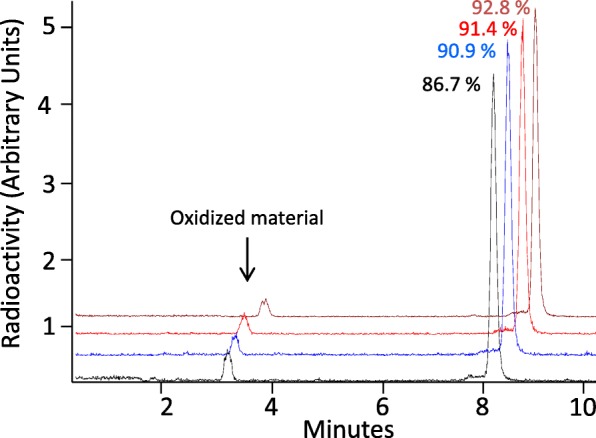
HPLC radiochemical purity analysis of [^68^Ga]Ga-DOTA-CP04 with no stabilizers in reactions mixture (black), with L-methionine as the only stabilizer (blue), with L-methionine, EtOH and sodium ascorbate as stabilizers (red) and with L-methionine, EtOH, sodium ascorbate and gentisic acid as stabilizers (brown)

Typical radiochemical purity of [^68^Ga]Ga-DOTA-CP04 determined by HPLC was 92–94%. The major radioactive impurities encountered in the production of [^68^Ga]Ga-DOTA-CP04 resulted from the oxidation of methionine to afford the sulfoxide analogue. Combined free and colloidal [^68^Ga]Ga was always ≤2%. In the absence of stabilisers, the purity of [^68^Ga]Ga-DOTA-CP04 was found to be 86.7%. Addition of a 10 mg/ml L-methionine solution (100 μL) as described for [^111^In]In-labelled CCK-2 targeting peptides improved the purity to 90.9%. In the presence of ethanol (200 μL), sodium ascorbate (200 μL of 0.05 M) and L-methionine (100 μL of 10 mg/ml) the purity increased marginally to 91.4% (Breeman et al., 2008). A combination of the above-mentioned stabilisers and 5-dihydroxybenzoic acid sodium salt (200 μL of 0.05 M) resulted in the optimal radiochemical purity achieved (92–94%) (Fig. 2).

RadioTLC analysis was performed using 1 M NH_4_OAc in methanol (1:1) as mobile phase according to the recommended conditions reported in the European Pharmacopeia monographs for the analysis of (^68^Ga) Edotreotide injection and Gallium (^68^Ga) chloride solution (European Directorate for the Quality of Medicines & Healthcare (EDQM), 2013a; European Directorate for the Quality of Medicines & Healthcare (EDQM), 2013b). Utilizing these conditions radiochemical purity was consistently ≥98% referring to the high purity of the [^68^Ga]Ga intact peptide with minimal amounts (≤2%) of free and colloidal [^68^Ga]Ga (Table 1).

**Table 1:** Pre-set acceptance criteria of [^68^Ga]Ga-DOTA-CP04 injection and the observed results

+Parameter	Specification	[^68^Ga]Ga-DOTA-CP04
Appearance	Clear and colorless	Pass
pH	4–8	5–6
Radionuclidic identity (half-life)	62–74 min	Pass
Radiochemical identity (HPLC)	Reference standard ±1.0 min	Pass
Radiochemical Purity (HPLC)	≥ 90% [^68^Ga]Ga-DOTA-CP04 < 8% oxidized material	92–94% 4–6%
Radiochemical Purity (TLC)	≥ 98% ≤ 2% free Ga-68	> 98% < 2%
Ethanol content	≤10%	9–10%
Bubbling point test	≥ 55 psi	Pass
Sterility	Sterile	No growth
Endotoxin	< 175 IU per dose	< 5 IU per dose

## Discussion

**Fig. 3:**
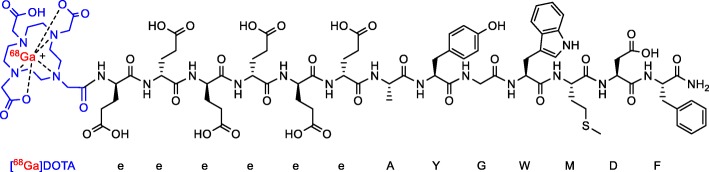
Chemical structure of [^68^Ga]Ga-DOTA-CP04

Cholecystokinin-2 (CCK-2) receptors represent an important molecular target overexpressed on a range of cancers, and particularly neuroendocrine tumor (NET) with low or absent somatostatin receptor subclass-2 (SSTR-2) expression. Based on pathological assessment, CCK-2 receptors are most commonly expressed by MTC (over 90% incidence) (Behr & Béhé, 2002; Gotthardt et al., 2006; Reubi et al., 1997), stromal ovarian cancer (100% incidence) (Reubi et al., 1997), Insulinoma (Körner et al., 2010) and small cell lung cancer (56% incidence) (Gotthardt et al., 2006; Reubi et al., 1997). Consequently, [^68^Ga]Ga-DOTA-CP04 (Fig. 3) has emerged as a potentially important radiopharmaceutical for staging such tumors, particularly in the context of elevated serum biomarkers like calcitonin in MTC or with equivocal conventional imaging. (Kunikowska et al., 2016). Indeed using [^111^In]In-DOTA-CP04 scintigraphy, 54.5% of all NET patients with negative SSTR-2 expression were found to overexpress CCK-2 receptors. This further confirms the potential role for [^68^Ga]Ga-DOTA-CP04 PET imaging in managing NET patients. As such, it is useful to develop a comprehensive automated production for [^68^Ga]Ga-DOTA-CP04 in hospital environments.

In our facility, the ^68^Ge/^68^Ga generator is only used for human production if [^68^Ge]germanium breakthrough content was below 0.005% as determined by measuring any residual long-lived radionuclidic contaminants in the eluate. Furthermore, any breakthrough of ^68^Ge is removed during the synthesis and the SPE purification to afford products with less than 0.001% [^68^Ge]germanium. The produced ^68^Ga-radiopharmaceuticals are tested individually to examine radiochemical identity, radiochemical purity, radionuclidic identity (half-life determination), pH and appearance. Acceptance criteria are set in accordance with European Pharmacopoeia (derived from monographs published for (^68^Ga) Edotreotide injection and Gallium (^68^Ga) chloride solution for radiolabeling (European Directorate for the Quality of Medicines & Healthcare (EDQM), 2013a; European Directorate for the Quality of Medicines & Healthcare (EDQM), 2013b) and international standards where applicable (Banerjee & Pomper, 2013; Vis et al., 2015; Velikyan, 2015). The suitability of the HPLC system (system suitability) to analyse the quality of radiopharmaceuticals produced is verified prior to any production using a corresponding reference standard material. This is essential to ascertain that the analytical system is fit for intended use (Zigler & New, 2008). Correlation of the retention time between the reference standard and the corresponding radiopharmaceutical is used to ascertain radiochemical identity.

The MultiSyn is a compact and versatile disposable cassette radiosynthesizer suited to the radiolabeling of theranostic agents. It consists of four 3-stopcock manifolds rotated by three-way position actuators, two syringe drives, built-in vacuum pump for solvent evaporation or reagent transfers and three tungsten collimated radioactivity detectors for synthesis monitoring. The disposable cassette materials have been carefully selected to minimize metal contamination.

Only three manifolds (M2–M4) are utilized for the production of ^68^Ga-labelled radiopharmaceuticals using our current reported method. [^68^Ga]Gallium is eluted using syringe 1 directly into the reaction vial for radiolabeling. It is possible that a fourth manifold (at M1) could be utilized to trap [^68^Ga]Ga prior to labelling if required by the use of different ^68^Ge/^68^Ga generators. In our current facility, the [^68^Ga]Ga eluate is delivered into the reactor via actuator 7 on Manifold 3 (M3). Manifold 4 (M2) is used to deliver ethanol, saline and water for product dilution, washing and formulation. Manifold 2 (M2) is utilized for SPE cartridge purification and formulation of the ^68^Ga-labelled radiopharmaceuticals. Nitrogen gas is used as the vehicle gas to transfer liquids and is filtered through a 0.2 μm Millex-FG filter placed between the Gas 2 (G2) outlet and the cassette. Figure 1 represents a schematic for the MultiSyn module.

**Fig. 4:**
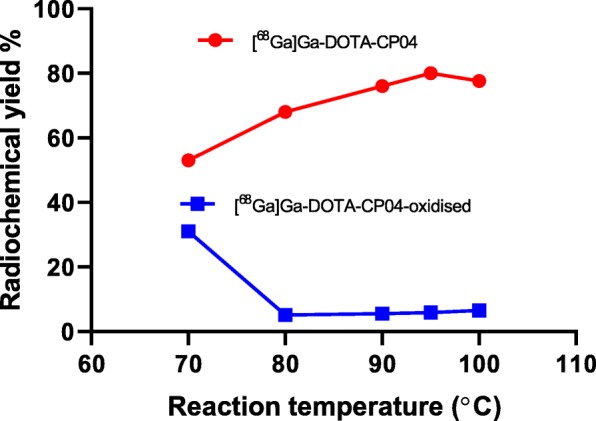
Effect of reaction temperature on formation yield of [^68^Ga]Ga-DOTA-CP04 (red) and its oxidized byproduct [^68^Ga]Ga-DOTA-CP04-oxidised (blue)

We screened the influence of reaction temperature from 70 to 105 °C on reactivity of DOTA-CP04 and the purity of the [^68^Ga]Ga-DOTA-CP04 product. Reaction performed at 95 °C afforded optimal yield of [^68^Ga]Ga-DOTA-CP04 and minimal formation of the oxidized material. Interestingly lowering the labelling temperature to 70 °C not only resulted in 20% reduction in the yield of [^68^Ga]Ga-DOTA-CP04 and increased free [^68^Ga]Ga, we also observed significantly increased oxidized material (Fig. 4). The radiochemical purity of [^68^Ga]Ga-DOTA-CP04 generated at 70 °C was only 65%. Furthermore, DOTA-CP04 required stronger buffering (0.5 M *c.f.* 0.25 M sodium acetate used for other radiopharmaceuticals in house) to facilitate labelling, presumably due to the acidic N-terminal hexaglutamate sequence of the peptide. The combination of stabilisers, sodium acetate solution, peptide and 0.05 M HCl (4 ml) used in the reaction mixture was verified to have pH 4.5.

**Fig. 5:**
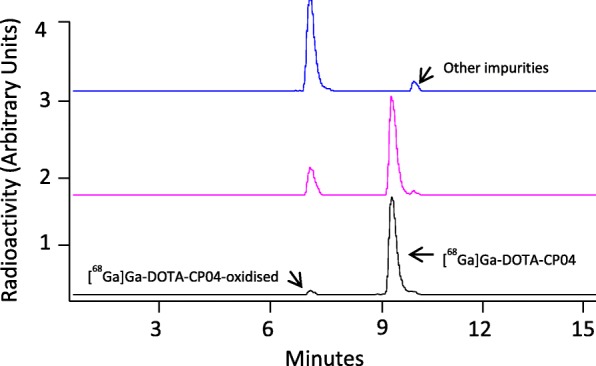
Addition of varying amounts of chloramine-T oxidant to [^68^Ga]Ga-DOTA-CP04 increases % formation of [^68^Ga]Ga-DOTA-CP04-oxidised material. Black chromatogram represents initial constituents of [^68^Ga]Ga-DOTA-CP04 for injection (93.8% pure) without the addition of any oxidants. [^68^Ga]Ga-DOTA-CP04 purity is reduced to 76.8% and the oxidized material increases to 21.14% in the presence of 0.439 μM chloramine-T oxidant (pink chromatogram). In the presence of 4.39 μM chloramine-T oxidant, [^68^Ga]Ga-DOTA-CP04 is completely consumed to form the oxidized material (93.4%) and one other impurity eluting directly after [^68^Ga]Ga-DOTA-CP04

Using the automated iPHASE MultiSyn module described above, the radiochemical purity of [^68^Ga]Ga-DOTA-CP04 was found to be 92–94% under the optimal labelling conditions with the major radiochemical impurity arising from the radiolytic oxidation of the methionine residue to the corresponding sulfoxide adduct. The formation of the sulfoxide adduct as the major radiochemical impurity has been demonstrated in prior work (Breeman et al., 2008). Further, we incubated small portions of the [^68^Ga]Ga-DOTA-CP04 for injection with varying amounts of chloramine-T oxidant (0.439–4.39 μM). A low concentration of chloramine-T (0.439 μM) reduced the radiochemical purity of [^68^Ga]Ga-DOTA-CP04 to 76.8% and increased the concentration of the oxidized material to 21.14%. At a higher concentration of chloramine-T (4.39 μM), [^68^Ga]Ga-DOTA-CP04 was completely consumed to form the oxidized material (93.4%)(Fig. 5).

Addition of numerous stabilisers including ethanol, sodium ascorbate, 2,5-dihydroxybenzoic acid and L-methionine resulted in the optimal and reproducible radiochemical purity of 92–94%. Accordingly, considering the nature of the peptide and the existence of the oxidation-prone methionine, we set the acceptance criteria for the radiochemical purity at ≥90% in line with other CP04 based radiopharmaceuticals (Maina et al., 2016; Pawlak et al., 2016). Moreover, there is precedence for having radiochemical purities of ≥90% as the cut-off criteria for many other radiopharmaceuticals used in humans. Ethanol, sodium ascorbate and 2,5-dihydroxybenzoic acid are common stabilizers used to prepare human-grade injectables in the radiopharmaceutical industry. L-methionine has been used in CP04 based radiopharmaceutical preparations to be used in first-in-human clinical trial (Maina et al., 2016; Pawlak et al., 2016). Moreover, L-methionine is a recommended stabilizer used in the preparation of parenteral injection of pharmaceuticals and has been used to treat liver disorders and lower urinary pH. Its LD_50_ in rats (IP administration) is 4.328 g/Kg (Rowe et al., 2003). It has been used in the formulation of the pharmaceutical Granocyte (intravenous and subcutaneous injections) at 1 mg/dose injections (Lipiäinen et al., 2015). Our final formulation contains no more than 0.01 mg/ml. As such, the final formulation of [^68^Ga]Ga-DOTA-CP04 is suitable for human injection.

The production protocol described herein utilizes ethanol as the only organic solvent, which is an excipient in the final formulation. It is essential to limit total ethanol in the end formulation to ≤10%. Careful development of the automated process ensured that less than 1.0 ml of ethanol is utilized to elute the ^68^Ga-labelled radiopharmaceuticals from the C-18 cartridge (Strata X). With subsequent addition of 9 ml saline, the final formulation of the ^68^Ga-labelled radiopharmaceuticals constitutes ≤10% ethanol content. Gas chromatography (GC) testing for ethanol content in [^68^Ga]Ga-DOTA-CP04 for injection confirmed that ethanol was consistently ≤10% of the final formulation.

## Conclusion

In conclusion, we have presented a controlled procedure for the automated production of ^68^Ga-labelled radiopharmaceuticals, specifically; [^68^Ga]Ga-DOTA-CP04 with the iPHASE MultiSyn module. This can be also adapted for the production of other human-grade ^68^Ga-labelled radiopharmaceuticals. All products passed pre-determined acceptance criteria and were prepared in a reproducible and efficient manner. Currently, we are investigating the utility of the MultiSyn module by applying it to the preparation of other ^68^Ga-labelled radiopharmaceuticals and other radiometal-labeled radiopharmaceuticals.

## Additional file

Additional file 1: **Figure S1:** Image of the iPHASE MultiSyn radiochemistry module. **Figure S2.** Radio-HPLC of [68Ga]Ga-DOTA-CP04 for injection. Chromatographic conditions: Kinetex XB C18 column (5 μm, 100 Å, 250 × 4.60 mm) eluted at 1 ml/min with a gradient of MeCN: 0.05% (v/v) TFA, starting at 25% MeCN for 1 min, increased to 90% B over 5 min and maintained at 90% MeCN for 10 min. **Figure S3.** Radio-TLC of [68Ga]Ga-DOTA-CP04 for injection. Spotted ITLC-SG trips were processed with aqueous 1 M NH4OAc in methanol (1:1) as mobile phase. **Figure S4.** LC-MS of DOTA-CP04 precursor. Solvent front contains sodium acetate salts as the precursor was dissolved in 0.5 M sodium acetate. **Figure S5.** MS/MS fragmentation profile of DOTA-CP04 precursor. **Figure S6.** A copy of the Multisyn Recipe. (PDF 1129 kb)

## Data Availability

Additional data is presented in the Additional file. Please contact the author for any additional data request.

## Abbreviations

[^68^Ga]Ga: [^68^Ga]Gallium
CCK-2R: Cholecystokinin-2 receptors
Co-57: Cobalt-57
Cs-157: Cesium-157
GMP: Good manufacturing practice
HCl: Hydrochloric acid
HPLC: High performance liquid chromatography
LC-MS: Liquid chromatography–mass spectrometry
Met: Methionine
MS/MS: Tandem mass spectrometry
NETs: Neuroendocrine tumors
PET: Positron emission tomography
SSTR-2: Somatostatin receptors
TLC: Thin layer chromatography
