# Supplementary material for: Automated preparation of clinical grade [68Ga]Ga-DOTA-CP04, a cholecystokinin-2 receptor agonist, using iPHASE MultiSyn synthesis platform
Source: EJNMMI Radiopharm Chem. 2019 Aug 23;4:23. doi: 10.1186/s41181-019-0067-2 (PMC6707997; doi:10.1186/s41181-019-0067-2)
Supplement: Supplementary file 1 — Figure S1: Image of the iPHASE MultiSyn radiochemistry module. Figure S2. Radio-HPLC of [68Ga]Ga-DOTA-CP04 for injection. Chromatographic conditions: Kinetex XB C18 column (5 μm, 100 Å, 250 × 4.60 mm) eluted at 1 ml/min with a gradient of MeCN: 0.05% (v/v) TFA, starting at 25% MeCN for 1 min, increased to 90% B over 5 min and maintained at 90% MeCN for 10 min. Figure S3. Radio-TLC of [68Ga]Ga-DOTA-CP04 for injection. Spotted ITLC-SG trips were processed with aqueous 1 M NH4OAc in methanol (1:1) as mobile phase. Figure S4. LC-MS of DOTA-CP04 precursor. Solvent front contains sodium acetate salts as the precursor was dissolved in 0.5 M sodium acetate. Figure S5. MS/MS fragmentation profile of DOTA-CP04 precursor. Figure S6. A copy of the Multisyn Recipe. (PDF 1129 kb) [file 41181_2019_67_MOESM1_ESM.pdf]

Automated preparation of clinical grade [^{68}Ga]Ga-DOTA-CP04, a cholecystokinin-2 receptor agonist, using a prototype synthesis platform (iPHASE MultiSyn)

Mohammad B. Haskali, [1,2] Peter D. Roselt, [1] David Binns, [1] Amit Hetsron, [1] Stan Poniger,[3] Craig A. Hutton [4,5] Rodney J. Hicks[1,2]

[1] The Centre for Molecular Imaging and Translational Research Laboratory, The Peter MacCallum Cancer Centre, Melbourne, Victoria, Australia and [2]Sir Peter MacCallum Department of Oncology, The University of Melbourne, Victoria 3010, Australia

[3] iPHASE Technologies Pty. Ltd. Melbourne, Australia.

[4] School of Chemistry and [5] Bio21 Molecular Science and Biotechnology Institute, The University of Melbourne, VIC 3010, Australia

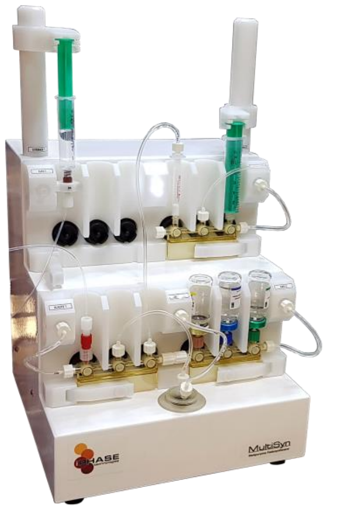

Figure S1: Image of the iPHASE MultiSyn radiochemistry module.

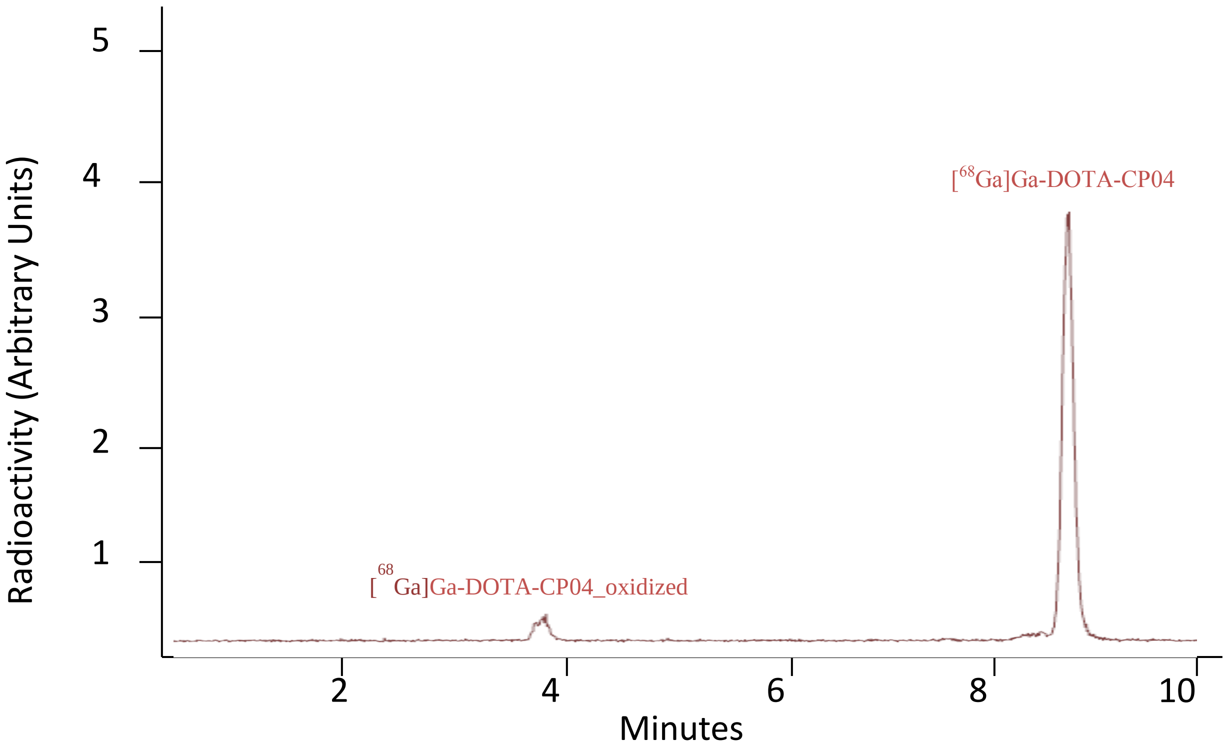

Figure S2: Radio-HPLC of [^{68}Ga]Ga-DOTA-CP04 for injection. Chromatographic conditions: Kinetex XB C18 column (5 µm, 100 Å, 250 × 4.60 mm) eluted at 1 ml/min with a gradient of MeCN: 0.05% (v/v) TFA, starting at 25% MeCN for 1 min, increased to 90% B over 5 min and maintained at 90% MeCN for 10 min.

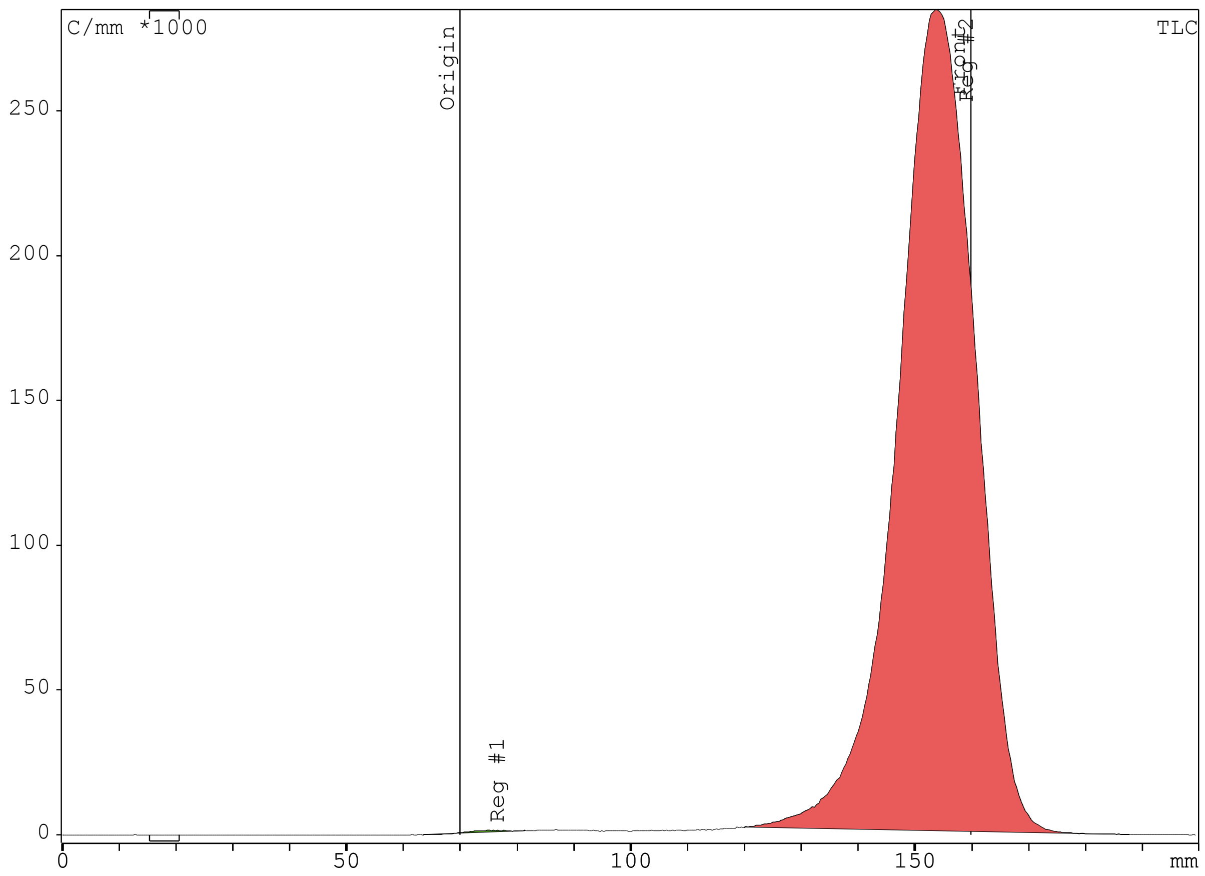

Figure S3: Radio-TLC of [^{68}Ga]Ga-DOTA-CP04 for injection. Spotted ITLC-SG trips were processed with aqueous 1 M NH_4OAc in methanol (1:1) as mobile phase.

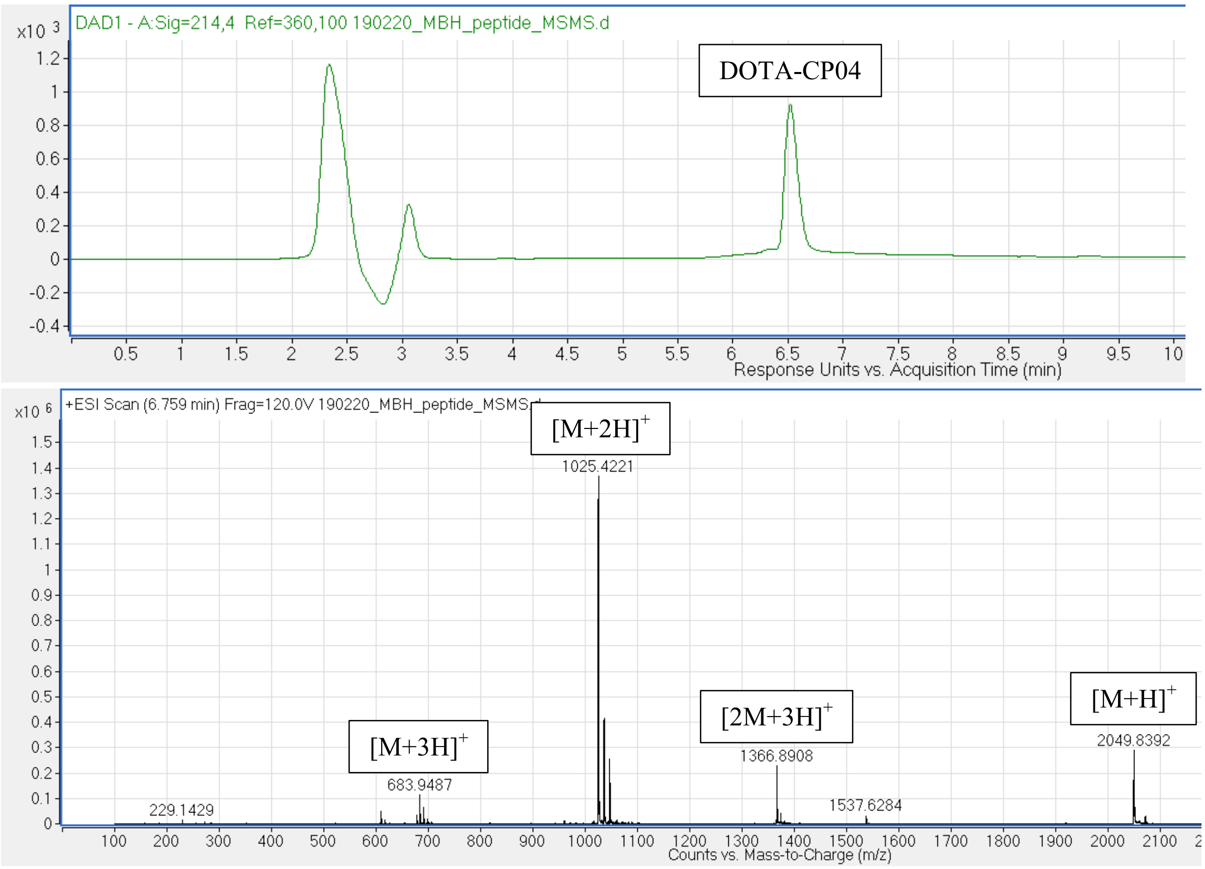

Figure S4: LC-MS of DOTA-CP04 precursor. Solvent front contains sodium acetate salts as the precursor was dissolved in 0.5M sodium acetate.

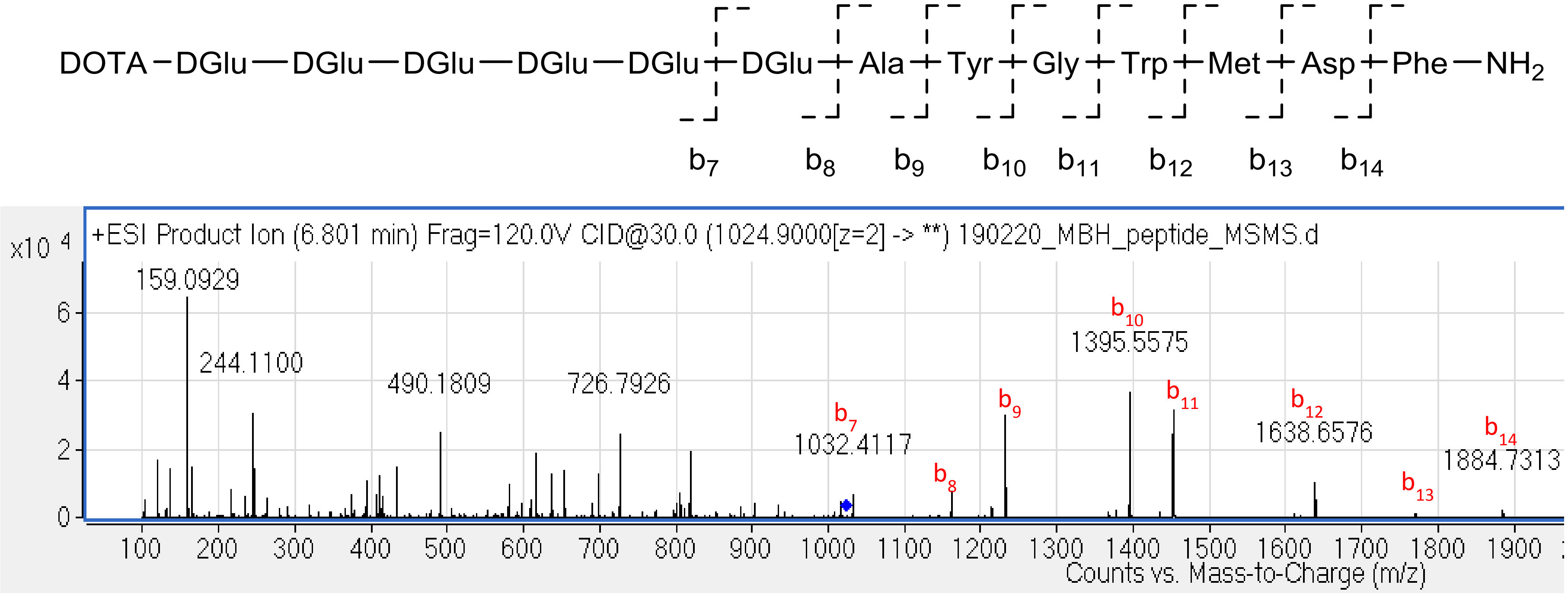

Figure S5: MS/MS fragmentation profile of DOTA-CP04 precursor.

tep	Step Message	Condition For Next Step	Step Time/Condition Timeout (sec)	RT1 (0=off, 1=left, 2=right)	RT2 (0=off, 1=left, 2=right)	RT3 (0=off, 1=left, 2=right)	RT4 (0=off, 1=left, 2=right)	RT5 (0=off, 1=left, 2=right)	RT6 (0=off, 1=left, 2=right)	RT7 (0=off, 1=left, 2=right)	RT8 (0=off, 1=left, 2=right)	RT9 (0=off, 1=left, 2=right)	RT10 (0=off, 1=left, 2=right)	RT11 (0=off, 1=left, 2=right)	RT12 (0=off, 1=left, 2=right)	Syringe 1 Position (0-25mL)	Syringe 2 Position (0-25mL)	V01 (0=off, 1=on)	V02 (0=off, 1=on)	V03 (0=off, 1=on)	V04 (0=off, 1=on)	V05 (0=off, 1=on)	V06 (0=off, 1=on)	V07 (0=off, 1=on)	V08 (0=off, 1=on)	Reactor 1 Temp (0-220 °C)	Vacuum Setpoint (-1.0-0.0 bar)	Prog. Output 1 (0=off, 1=on)	Prog. Output 2 (0=off, 1=on)	Prog. Output 3 (0=off, 1=on)
1	Remove old hardware kit - Click NEXT when done	0	0	0	0	0	0	0	0	0	0	0	0	0	0	0.0	0.1	0	0	0	0	0	0	0	0	0.0	0.00	0	0	0
2	Remove HCl syringe 1 - Click NEXT when done	0	0	0	0	0	0	0	0	0	0	0	0	0	0	0.0	0.1	0	0	0	0	0	0	0	0	0.0	0.00	0	0	0
3	Install new Ga-68 hardware kit (no reagents) - Click NEXT whe	0	0	0	0	0	0	0	0	0	0	0	0	0	0	0.0	0.1	0	0	0	0	0	0	0	0	0.0	0.00	0	0	0
4	Pressure testing gas connection to manifold 4	0	2	0	0	0	0	0	0	0	0	0	0	0	0	0.0	0.1	1	0	1	0	0	0	0	1	0.0	-0.10	0	0	0
5	Pressure testing gas connection to manifold 4	0	5	0	0	0	0	0	0	0	0	0	0	0	1	0.0	0.1	1	0	1	0	0	0	0	0	0.0	0.00	0	0	0
6	Pressure testing gas connection to manifold 4	0	9	0	0	0	0	0	0	0	0	0	0	0	1	0.0	0.1	0	0	1	0	0	0	0	0	0.0	0.00	0	0	0
7	Pressure testing gas connection to manifold 4	20	1	0	0	0	0	0	0	0	0	0	0	0	1	0.0	0.1	0	0	1	0	0	0	0	0	0.0	0.00	0	0	0
8	Pressure testing connection between manifold 4 and 3	0	2	0	0	0	0	0	0	0	0	0	0	0	0	0.0	0.1	1	0	1	0	0	0	0	1	0.0	-0.10	0	0	0
9	Pressure testing connection between manifold 4 and 3	0	5	0	0	0	0	0	0	0	0	1	0	0	0	0.0	0.1	1	0	1	0	0	0	0	0	0.0	0.00	0	0	0
10	Pressure testing connection between manifold 4 and 3	0	9	0	0	0	0	0	0	0	0	1	0	0	0	0.0	0.1	0	0	1	0	0	0	0	0	0.0	0.00	0	0	0
11	Pressure testing connection between manifold 4 and 3	20	1	0	0	0	0	0	0	0	0	1	0	0	0	0.0	0.1	0	0	1	0	0	0	0	0	0.0	0.00	0	0	0
12	Pressure testing reactor connection	0	2	0	0	0	0	0	0	0	2	0	0	0	0	0.0	0.1	1	0	1	0	0	0	1	0	0.0	-0.10	0	0	0
13	Pressure testing reactor connection	0	7	0	0	0	0	0	0	0	2	0	0	0	0	0.0	0.1	1	0	1	0	0	0	0	0	0.0	0.00	0	0	0
14	Pressure testing reactor connection	0	9	0	0	0	0	0	0	0	2	0	0	0	0	0.0	0.1	0	0	1	0	0	0	0	0	0.0	0.00	0	0	0
15	Pressure testing reactor connection	20	1	0	0	0	0	0	0	0	2	0	0	0	0	0.0	0.1	0	0	1	0	0	0	0	0	0.0	0.00	0	0	0
16	Pressure testing waste connection to manifold 3	0	2	0	0	0	0	0	0	0	0	0	0	0	0	0.0	0.1	1	0	1	0	0	0	0	1	0.0	-0.10	0	0	0
17	Pressure testing waste connection to manifold 3	0	5	0	0	0	0	0	0	0	0	0	0	0	0	0.0	0.1	1	0	1	0	0	0	0	0	0.0	0.00	0	0	0
18	Pressure testing waste connection to manifold 3	0	9	0	0	0	0	0	0	0	0	0	0	0	0	0.0	0.1	0	0	1	0	0	0	0	0	0.0	0.00	0	0	0
19	Pressure testing waste connection to manifold 3	20	1	0	0	0	0	0	0	0	0	0	0	0	0	0.0	0.1	0	0	1	0	0	0	0	0	0.0	0.00	0	0	0
20	Pressure testing SPE cartridge between manfold 3 and 2	0	2	0	0	0	2	0	0	0	0	2	0	0	0	0.0	0.1	1	0	1	0	1	0	0	0	0.0	-0.10	0	0	0
21	Pressure testing SPE cartridge between manfold 3 and 2	0	5	0	0	0	0	0	0	0	0	2	0	0	0	0.0	0.1	1	0	1	0	0	0	0	0	0.0	0.00	0	0	0
22	Pressure testing SPE cartridge between manfold 3 and 2	0	9	0	0	0	0	0	0	0	0	2	0	0	0	0.0	0.1	0	0	1	0	0	0	0	0	0.0	0.00	0	0	0
23	Pressure testing SPE cartridge between manfold 3 and 2	20	1	0	0	0	0	0	0	0	0	2	0	0	0	0.0	0.1	0	0	1	0	0	0	0	0	0.0	0.00	0	0	0
24	Pressure testing syringe 2 connection	0	2	0	0	0	2	0	0	0	0	2	0	0	0	0.0	0.1	1	0	1	0	1	0	0	0	0.0	-0.10	0	0	0
25	Pressure testing syringe 2 connection	0	5	0	0	0	2	0	1	0	0	2	0	0	0	0.0	0.1	1	0	1	0	0	0	0	0	0.0	0.00	0	0	0
26	Pressure testing syringe 2 connection	0	9	0	0	0	2	0	1	0	0	2	0	0	0	0.0	0.1	0	0	1	0	0	0	0	0	0.0	0.00	0	0	0
27	Pressure testing syringe 2 connection	20	1	0	0	0	2	0	1	0	0	2	0	0	0	0.0	0.1	0	0	1	0	0	0	0	0	0.0	0.00	0	0	0
28	Pressure testing waste connection to manifold 2	0	2	0	0	0	2	0	0	0	0	2	0	0	0	0.0	0.1	1	0	1	0	1	0	0	0	0.0	-0.10	0	0	0
29	Pressure testing waste connection to manifold 2	0	5	0	0	0	2	0	0	0	0	2	0	0	0	0.0	0.1	1	0	1	0	0	0	0	0	0.0	-0.10	0	0	0
30	Pressure testing waste connection to manifold 2	0	9	0	0	0	2	0	0	0	0	2	0	0	0	0.0	0.1	0	0	1	0	0	0	0	0	0.0	-0.10	0	0	0
31	Pressure testing waste connection to manifold 2	20	1	0	0	0	2	0	0	0	0	2	0	0	0	0.0	0.1	0	0	1	0	0	0	0	0	0.0	-0.10	0	0	0
32	Flushing water vial spike	0	2	0	0	0	0	0	0	0	0	0	0	0	2	0.0	0.1	1	0	1	0	0	0	0	1	0.0	-0.10	0	0	0
33	Flushing saline vial spike	0	2	0	0	0	0	0	0	0	0	0	0	2	0	0.0	0.1	1	0	1	0	0	0	0	1	0.0	-0.10	0	0	0
34	Flushing ethanol vial spike	0	2	0	0	0	0	0	0	0	0	0	2	0	0	0.0	0.1	1	0	1	0	0	0	0	1	0.0	-0.10	0	0	0
35	Flushing manifolds 3 and 4	0	2	0	0	0	0	0	0	0	0	0	0	0	0	0.0	0.1	1	0	1	0	0	0	0	1	0.0	-0.10	0	0	0

Step	Step Message	Condition For Next Step	Step Time/Condition Timeout (sec)	RT1 (0=off, 1=left, 2=right)	RT2 (0=off, 1=left, 2=right)	RT3 (0=off, 1=left, 2=right)	RT4 (0=off, 1=left, 2=right)	RT5 (0=off, 1=left, 2=right)	RT6 (0=off, 1=left, 2=right)	RT7 (0=off, 1=left, 2=right)	RT8 (0=off, 1=left, 2=right)	RT9 (0=off, 1=left, 2=right)	RT10 (0=off, 1=left, 2=right)	RT11 (0=off, 1=left, 2=right)	RT12 (0=off, 1=left, 2=right)	Syringe 1 Position (0-25mL)	Syringe 2 Position (0-25mL)	V01 (0=off, 1=on)	V02 (0=off, 1=on)	V03 (0=off, 1=on)	V04 (0=off, 1=on)	V05 (0=off, 1=on)	V06 (0=off, 1=on)	V07 (0=off, 1=on)	V08 (0=off, 1=on)	Reactor 1 Temp (0-220 °C)	Vacuum Setpoint (-1.0-0.0 bar)	Prog. Output 1 (0=off, 1=on)	Prog. Output 2 (0=off, 1=on)	Prog. Output 3 (0=off, 1=on)
36	Flushing reactor	0	10	0	0	0	0	0	0	0	2	0	0	0	0	0.0	0.1	1	0	1	0	0	0	1	0	0.0	-0.10	0	0	0
37	Flushing SPE cartridge	0	10	0	0	0	2	0	0	0	0	2	0	0	0	0.0	0.1	1	0	1	0	1	0	0	0	0.0	-0.10	0	0	0
38	Connect 70% Ethanol vial to product out to sterilize product de	0	0	0	0	0	0	0	0	0	0	0	0	0	0	0.0	0.1	0	0	0	0	0	0	0	0	0.0	0.00	0	0	0
39	Flush 70% Ethanol to sterilize product delivery line	0	80	0	0	0	2	1	0	0	0	2	0	0	0	0.0	0.1	1	0	1	0	0	0	0	0	0.0	0.00	0	0	0
40	Connect product out directly to product delivery line - Click N	0	0	0	0	0	0	0	0	0	0	0	0	0	0	0.0	0.1	0	0	0	0	0	0	0	0	0.0	0.00	0	0	0
41	Flush gas through product delivery line	0	240	0	0	0	2	1	0	0	0	2	0	0	0	0.0	0.1	1	0	1	0	0	0	0	0	0.0	0.00	0	0	0
42	Install all reagents - Click NEXT when done	0	0	0	0	0	0	0	0	0	0	0	0	0	0	4.0	0.1	0	0	0	0	0	0	1	0	0.0	0.00	0	0	0
43	Inject precursor solution into reactor centre port - Click NEXT	0	0	0	0	0	0	0	0	0	0	0	0	0	0	4.0	0.1	0	0	0	0	0	0	1	0	0.0	-0.05	0	0	0
44	Connect 4mL 0.05M HCl syringe 1 and Ga-68 generator - Clic	0	0	0	0	0	0	0	0	0	0	0	0	0	0	4.0	0.1	0	0	0	0	0	0	0	0	0.0	0.00	0	0	0
45	Connect product collection vial - Click NEXT when done	0	0	0	0	0	0	0	0	0	0	0	0	0	0	4.0	0.1	0	0	0	0	0	0	0	0	0.0	0.00	0	0	0
46	Pressurizing ethanol vial	0	8	0	0	0	0	0	0	0	0	0	2	0	0	4.0	0.1	1	0	1	0	0	0	0	0	0.0	-0.10	0	0	0
47	Pressurizing saline vial	0	8	0	0	0	0	0	0	0	0	0	0	2	0	4.0	0.1	1	0	1	0	0	0	0	0	0.0	-0.10	0	0	0
48	Pressurizing water vial	0	8	0	0	0	0	0	0	0	0	0	0	0	2	4.0	0.1	1	0	1	0	0	0	0	0	0.0	-0.10	0	0	0
49	De-pressurizing SPE cartridge	0	4	0	0	0	2	0	0	0	0	2	0	0	0	4.0	0.1	0	0	1	0	1	0	0	0	0.0	-0.10	0	0	0
50	Conditioning SPE cartridge with ethanol	43	60	0	0	0	2	0	1	0	0	2	1	0	0	4.0	2.2	0	0	0	0	0	0	0	0	0.0	-0.10	0	0	0
51	Conditioning SPE cartridge with ethanol	0	10	0	0	0	2	0	1	0	0	2	1	0	0	4.0	2.2	0	0	0	0	0	0	0	0	0.0	-0.10	0	0	0
52	Conditioning SPE cartridge with water	43	60	0	0	0	2	0	1	0	0	2	0	0	1	4.0	6.0	0	0	0	0	0	0	0	0	0.0	-0.10	0	0	0
53	Conditioning SPE cartridge with water	0	30	0	0	0	2	0	1	0	0	2	0	0	1	4.0	6.0	0	0	0	0	0	0	0	0	0.0	-0.10	0	0	0
54	De-pressurizing kit	0	2	0	0	0	2	0	1	0	0	0	0	0	0	4.0	6.0	0	0	1	0	0	0	0	1	0.0	-0.10	0	0	0
55	Pressurizing water vial	0	8	0	0	0	2	0	1	0	0	0	0	0	2	4.0	6.0	1	0	1	0	0	0	0	0	0.0	-0.10	0	0	0
56	De-pressurizing kit	0	2	0	0	0	2	0	1	0	0	0	0	0	0	4.0	6.0	0	0	1	0	0	0	0	1	0.0	-0.10	0	0	0
57	Conditioning SPE cartridge with water	43	60	0	0	0	2	0	1	0	0	2	0	0	1	4.0	10.0	0	0	0	0	0	0	0	0	0.0	-0.10	0	0	0
58	Conditioning SPE cartridge with water	0	30	0	0	0	2	0	1	0	0	2	0	0	1	4.0	10.0	0	0	0	0	0	0	0	0	0.0	-0.10	0	0	0
59	Pressurize syringe 2	0	10	0	0	0	2	0	1	0	0	2	0	0	0	4.0	12.0	1	0	1	0	0	0	0	0	0.0	-0.10	0	0	0
60	Emptying syringe 2 and re-pressurizing water vial	43	60	0	0	0	2	0	2	0	0	2	0	0	2	4.0	0.1	1	0	1	0	1	0	0	0	0.0	-0.10	0	0	0
61	Emptying syringe 2	0	5	0	0	0	2	0	2	0	0	2	0	0	0	4.0	0.0	1	0	1	0	1	0	0	0	0.0	-0.10	0	0	0
62	Flushing SPE cartridge	0	25	0	0	0	2	0	0	0	0	2	0	0	0	4.0	0.0	1	0	1	0	1	0	0	0	0.0	-0.10	0	0	0
63	De-pressurizing kit	0	3	0	0	0	2	0	0	0	0	2	0	0	0	4.0	0.0	0	0	1	0	1	0	0	0	0.0	0.00	0	0	0
64	Ready to elute Ga-68 generator - Press NEXT to start elution	1	0	0	0	0	0	0	0	0	0	0	0	0	0	4.0	0.0	0	0	0	0	0	0	0	0	0.0	-0.10	0	0	0
65	Eluting Ga-68 generator to reactor	42	30	0	0	0	0	0	0	2	1	0	0	0	0	3.7	0.0	0	0	0	0	0	0	1	0	90.0	-0.10	0	0	0
66	Eluting Ga-68 generator to reactor	0	4	0	0	0	0	0	0	2	1	0	0	0	0	3.7	0.0	0	0	0	0	0	0	1	0	90.0	-0.10	0	0	0
67	Eluting Ga-68 generator to reactor	42	30	0	0	0	0	0	0	2	1	0	0	0	0	3.4	0.0	0	0	0	0	0	0	1	0	90.0	-0.10	0	0	0
68	Eluting Ga-68 generator to reactor	0	4	0	0	0	0	0	0	2	1	0	0	0	0	3.4	0.0	0	0	0	0	0	0	1	0	90.0	-0.10	0	0	0
69	Eluting Ga-68 generator to reactor	42	30	0	0	0	0	0	0	2	1	0	0	0	0	3.1	0.0	0	0	0	0	0	0	1	0	90.0	-0.10	0	0	0
70	Eluting Ga-68 generator to reactor	0	4	0	0	0	0	0	0	2	1	0	0	0	0	3.1	0.0	0	0	0	0	0	0	1	0	90.0	-0.10	0	0	0

Step	Step Message	Condition For Next Step	Step Time/Condition Timeout (sec)	RT1 (0=off, 1=left, 2=right)	RT2 (0=off, 1=left, 2=right)	RT3 (0=off, 1=left, 2=right)	RT4 (0=off, 1=left, 2=right)	RT5 (0=off, 1=left, 2=right)	RT6 (0=off, 1=left, 2=right)	RT7 (0=off, 1=left, 2=right)	RT8 (0=off, 1=left, 2=right)	RT9 (0=off, 1=left, 2=right)	RT10 (0=off, 1=left, 2=right)	RT11 (0=off, 1=left, 2=right)	RT12 (0=off, 1=left, 2=right)	Syringe 1 Position (0-25mL)	Syringe 2 Position (0-25mL)	V01 (0=off, 1=on)	V02 (0=off, 1=on)	V03 (0=off, 1=on)	V04 (0=off, 1=on)	V05 (0=off, 1=on)	V06 (0=off, 1=on)	V07 (0=off, 1=on)	V08 (0=off, 1=on)	Reactor 1 Temp (0-220 °C)	Vacuum Setpoint (-1.0-0.0 bar)	Prog. Output 1 (0=off, 1=on)	Prog. Output 2 (0=off, 1=on)
71	Eluting Ga-68 generator to reactor	42	30	0	0	0	0	0	0	2	1	0	0	0	0	2.8	0.0	0	0	0	0	0	0	1	0	90.0	-0.10	0	0
72	Eluting Ga-68 generator to reactor	0	4	0	0	0	0	0	0	2	1	0	0	0	0	2.8	0.0	0	0	0	0	0	0	1	0	90.0	-0.10	0	0
73	Eluting Ga-68 generator to reactor	42	30	0	0	0	0	0	0	2	1	0	0	0	0	2.5	0.0	0	0	0	0	0	0	1	0	90.0	-0.10	0	0
74	Eluting Ga-68 generator to reactor	0	4	0	0	0	0	0	0	2	1	0	0	0	0	2.5	0.0	0	0	0	0	0	0	1	0	90.0	-0.10	0	0
75	Eluting Ga-68 generator to reactor	42	30	0	0	0	0	0	0	2	1	0	0	0	0	2.2	0.0	0	0	0	0	0	0	1	0	90.0	-0.10	0	0
76	Eluting Ga-68 generator to reactor	0	4	0	0	0	0	0	0	2	1	0	0	0	0	2.2	0.0	0	0	0	0	0	0	1	0	90.0	-0.10	0	0
77	Eluting Ga-68 generator to reactor	42	30	0	0	0	0	0	0	2	1	0	0	0	0	1.9	0.0	0	0	0	0	0	0	1	0	90.0	-0.10	0	0
78	Eluting Ga-68 generator to reactor	0	4	0	0	0	0	0	0	2	1	0	0	0	0	1.9	0.0	0	0	0	0	0	0	1	0	90.0	-0.10	0	0
79	Eluting Ga-68 generator to reactor	42	30	0	0	0	0	0	0	2	1	0	0	0	0	1.6	0.0	0	0	0	0	0	0	1	0	90.0	-0.10	0	0
80	Eluting Ga-68 generator to reactor	0	4	0	0	0	0	0	0	2	1	0	0	0	0	1.6	0.0	0	0	0	0	0	0	1	0	90.0	-0.10	0	0
81	Eluting Ga-68 generator to reactor	42	30	0	0	0	0	0	0	2	1	0	0	0	0	1.3	0.0	0	0	0	0	0	0	1	0	90.0	-0.10	0	0
82	Eluting Ga-68 generator to reactor	0	4	0	0	0	0	0	0	2	1	0	0	0	0	1.3	0.0	0	0	0	0	0	0	1	0	90.0	-0.10	0	0
83	Eluting Ga-68 generator to reactor	42	30	0	0	0	0	0	0	2	1	0	0	0	0	1.0	0.0	0	0	0	0	0	0	1	0	90.0	-0.10	0	0
84	Eluting Ga-68 generator to reactor	0	4	0	0	0	0	0	0	2	1	0	0	0	0	1.0	0.0	0	0	0	0	0	0	1	0	90.0	-0.10	0	0
85	Eluting Ga-68 generator to reactor	42	30	0	0	0	0	0	0	2	1	0	0	0	0	0.7	0.0	0	0	0	0	0	0	1	0	90.0	-0.10	0	0
86	Eluting Ga-68 generator to reactor	0	4	0	0	0	0	0	0	2	1	0	0	0	0	0.7	0.0	0	0	0	0	0	0	1	0	90.0	-0.10	0	0
87	Eluting Ga-68 generator to reactor	42	30	0	0	0	0	0	0	2	1	0	0	0	0	0.4	0.0	0	0	0	0	0	0	1	0	90.0	-0.10	0	0
88	Eluting Ga-68 generator to reactor	0	4	0	0	0	0	0	0	2	1	0	0	0	0	0.4	0.0	0	0	0	0	0	0	1	0	90.0	-0.10	0	0
89	Eluting Ga-68 generator to reactor	42	30	0	0	0	0	0	0	2	1	0	0	0	0	0.0	0.0	0	0	0	0	0	0	1	0	90.0	-0.10	0	0
90	Eluting Ga-68 generator to reactor	0	10	0	0	0	0	0	0	2	1	0	0	0	0	0.0	0.0	0	0	0	0	0	0	1	0	90.0	-0.10	0	0
91	Flushing HCl to reactor	0	5	0	0	0	0	0	0	0	0	0	0	0	0	0.0	0.0	0	1	1	0	0	0	1	1	90.0	-0.10	0	0
92	Flushing HCl to reactor	0	5	0	0	0	0	0	0	0	2	0	0	0	0	0.0	0.0	0	1	1	0	0	0	1	1	90.0	-0.10	0	0
93	Labelling reaction - Heat up phase	11	360	0	0	0	0	0	0	0	0	0	0	0	0	0.0	0.0	0	0	0	0	0	0	0	0	95.0	0.00	0	0
94	Labelling reaction - Heat up phase	0	60	0	0	0	0	0	0	0	0	0	0	0	0	0.0	0.0	0	0	0	0	0	0	0	0	95.0	0.00	0	0
95	Labelling reaction - Reaction time	0	420	0	0	0	0	0	0	0	0	0	0	0	0	0.0	0.0	0	0	0	0	0	0	0	0	95.0	0.00	0	0
96	Cooling down reactor	15	300	0	0	0	0	0	0	0	0	0	0	0	0	0.0	0.0	0	0	0	0	0	0	0	0	40.0	0.00	0	0
97	Trapping production onto SPE cartridge	0	70	0	0	0	2	0	0	0	2	1	0	0	0	0.0	0.0	0	1	0	0	1	1	0	0	0.0	-0.10	0	0
98	Depressurizing kit	0	5	0	0	0	2	0	0	0	2	1	0	0	0	0.0	0.0	0	0	0	0	1	0	0	0	0.0	0.00	0	0
99	Transferring 5mL water to reactor	0	8	0	0	0	2	0	0	0	2	0	0	0	1	0.0	0.0	0	0	0	0	0	0	1	0	0.0	-0.10	0	0
100	Washing SPE cartridge with water	0	30	0	0	0	2	0	0	0	2	1	0	0	0	0.0	0.0	1	0	0	0	1	1	0	0	0.0	-0.10	0	0
101	Flushing SPE cartridge	0	25	0	0	0	2	0	0	0	0	2	0	0	0	0.0	0.0	1	0	1	0	1	0	0	0	0.0	-0.10	0	0
102	De-pressurizing kit	0	3	0	0	0	2	0	0	0	0	2	0	0	0	0.0	0.0	0	0	1	0	1	0	0	0	0.0	0.00	0	0
103	Eluting SPE cartridge with ethanol	0	2	0	0	0	0	0	0	0	0	2	1	0	0	0.0	0.0	0	0	0	0	0	0	0	0	0.0	-0.10	0	0
104	Eluting SPE cartridge with ethanol	0	6	0	0	0	0	0	0	0	0	2	1	0	0	0.0	0.0	1	0	1	0	0	0	0	0	0.0	-0.10	0	0
105	Eluting SPE cartridge with ethanol	0	2	0	0	0	0	0	1	0	0	2	0	0	0	0.0	0.0	1	0	1	0	0	0	0	0	0.0	-0.10	0	0

Step	Step Message	Condition For Next Step	Step Time/Condition Timeout (sec)	RT1 (0=off, 1=left, 2=right)	RT2 (0=off, 1=left, 2=right)	RT3 (0=off, 1=left, 2=right)	RT4 (0=off, 1=left, 2=right)	RT5 (0=off, 1=left, 2=right)	RT6 (0=off, 1=left, 2=right)	RT7 (0=off, 1=left, 2=right)	RT8 (0=off, 1=left, 2=right)	RT9 (0=off, 1=left, 2=right)	RT10 (0=off, 1=left, 2=right)	RT11 (0=off, 1=left, 2=right)	RT12 (0=off, 1=left, 2=right)	Syringe 1 Position (0-25mL)	Syringe 2 Position (0-25mL)	V01 (0=off, 1=on)	V02 (0=off, 1=on)	V03 (0=off, 1=on)	V04 (0=off, 1=on)	V05 (0=off, 1=on)	V06 (0=off, 1=on)	V07 (0=off, 1=on)	V08 (0=off, 1=on)	Reactor 1 Temp (0-220 °C)	Vacuum Setpoint (-1.0-0.0 bar)	Prog. Output 1 (0=off, 1=on)	Prog. Output 2 (0=off, 1=on)
106	Eluting SPE cartridge with ethanol	43	30	0	0	0	2	0	1	0	0	2	0	0	0	0.0	0.5	0	0	0	0	0	0	0	0	0.0	-0.10	0	0
107	Eluting SPE cartridge with ethanol	0	5	0	0	0	2	0	1	0	0	2	0	0	0	0.0	0.5	0	0	0	0	0	0	0	0	0.0	-0.10	0	0
108	Eluting SPE cartridge with ethanol	43	30	0	0	0	2	0	1	0	0	2	0	0	0	0.0	1.0	0	0	0	0	0	0	0	0	0.0	-0.10	0	0
109	Eluting SPE cartridge with ethanol	0	5	0	0	0	2	0	1	0	0	2	0	0	0	0.0	1.0	0	0	0	0	0	0	0	0	0.0	-0.10	0	0
110	Eluting SPE cartridge with ethanol	43	30	0	0	0	2	0	1	0	0	2	0	0	0	0.0	1.5	0	0	0	0	0	0	0	0	0.0	-0.10	0	0
111	Eluting SPE cartridge with ethanol	0	5	0	0	0	2	0	1	0	0	2	0	0	0	0.0	1.5	0	0	0	0	0	0	0	0	0.0	-0.10	0	0
112	Eluting SPE cartridge with ethanol	43	30	0	0	0	2	0	1	0	0	2	0	0	0	0.0	2.0	0	0	0	0	0	0	0	0	0.0	-0.10	0	0
113	Eluting SPE cartridge with ethanol	0	5	0	0	0	2	0	1	0	0	2	0	0	0	0.0	2.0	0	0	0	0	0	0	0	0	0.0	-0.10	0	0
114	Diluting syringe 2 with saline	43	60	0	0	0	2	0	1	0	0	2	0	1	0	0.0	3.5	0	0	0	0	0	0	0	0	0.0	-0.10	0	0
115	Diluting syringe 2 with saline	0	35	0	0	0	2	0	1	0	0	2	0	1	0	0.0	3.5	0	0	0	0	0	0	0	0	0.0	-0.10	0	0
116	Pressurizing syringe 2	43	30	0	0	0	2	0	1	0	0	2	0	0	0	0.0	10.0	1	0	1	0	0	0	0	0	0.0	-0.10	0	0
117	Pressurizing syringe 2	0	14	0	0	0	2	0	1	0	0	2	0	0	0	0.0	10.0	1	0	1	0	0	0	0	0	0.0	-0.10	0	0
118	Transferring product out	43	60	0	0	0	2	2	1	0	0	2	0	0	0	0.0	5.0	0	0	0	0	0	0	0	0	0.0	-0.10	0	0
119	Transferring product out	0	5	0	0	0	2	2	1	0	0	2	0	0	0	0.0	5.0	0	0	0	0	0	0	0	0	0.0	-0.10	0	0
120	Transferring product out and re-pressurize saline vial	43	60	0	0	0	2	2	1	0	0	2	0	2	0	0.0	0.1	1	0	1	0	0	0	0	0	0.0	-0.10	0	0
121	Transferring product out and re-pressurize saline vial	0	5	0	0	0	2	2	1	0	0	2	0	2	0	0.0	0.0	1	0	1	0	0	0	0	0	0.0	-0.10	0	0
122	Flushing product out tubing	0	2	0	0	0	2	1	0	0	0	2	0	0	0	0.0	0.0	0	0	1	0	0	0	0	0	0.0	-0.10	0	0
123	Flushing product out tubing	0	10	0	0	0	2	1	0	0	0	2	0	0	0	0.0	0.0	0	1	1	0	0	0	0	0	0.0	-0.10	0	0
124	De-pressurizing kit	0	3	0	0	0	2	0	0	0	0	2	0	0	0	0.0	0.0	0	0	1	0	1	0	0	0	0.0	-0.10	0	0
125	Filling syringe 2 with saline	43	60	0	0	0	2	0	1	0	0	2	0	1	0	0.0	5.5	0	0	0	0	0	0	0	0	0.0	-0.10	0	0
126	Filling syringe 2 with saline	0	40	0	0	0	2	0	1	0	0	2	0	1	0	0.0	5.5	0	0	0	0	0	0	0	0	0.0	-0.10	0	0
127	De-pressurizing kit	0	2	0	0	0	2	0	1	0	0	0	0	0	0	0.0	5.5	0	0	1	0	0	0	0	1	0.0	-0.10	0	0
128	Re-pressurizing saline vial	0	8	0	0	0	2	0	1	0	0	0	0	2	0	0.0	5.5	1	0	1	0	0	0	0	0	0.0	-0.10	0	0
129	De-pressurizing kit	0	2	0	0	0	2	0	1	0	0	0	0	0	0	0.0	5.5	0	0	1	0	0	0	0	1	0.0	-0.10	0	0
130	Filling syringe 2 with saline	43	60	0	0	0	2	0	1	0	0	2	0	1	0	0.0	10.5	0	0	0	0	0	0	0	0	0.0	-0.10	0	0
131	Filling syringe 2 with saline	0	40	0	0	0	2	0	1	0	0	2	0	1	0	0.0	10.5	0	0	0	0	0	0	0	0	0.0	-0.10	0	0
132	Pressurizing syringe 2	43	30	0	0	0	2	0	1	0	0	2	0	0	0	0.0	11.5	1	0	1	0	0	0	0	0	0.0	-0.10	0	0
133	Pressurizing syringe 2	0	20	0	0	0	2	0	1	0	0	2	0	0	0	0.0	11.5	1	0	1	0	0	0	0	0	0.0	-0.10	0	0
134	Transferring saline rinse and dilution to product out	43	60	0	0	0	2	2	1	0	0	2	0	0	0	0.0	5.0	0	0	0	0	0	0	0	0	0.0	-0.10	0	0
135	Transferring saline rinse and dilution to product out	0	5	0	0	0	2	2	1	0	0	2	0	0	0	0.0	5.0	0	0	0	0	0	0	0	0	0.0	-0.10	0	0
136	Transferring saline rinse and dilution to product out	43	60	0	0	0	2	2	1	0	0	2	0	0	0	0.0	0.1	0	0	0	0	0	0	0	0	0.0	-0.10	0	0
137	Transferring saline rinse and dilution to product out	0	5	0	0	0	2	2	1	0	0	2	0	0	0	0.0	0.0	0	0	0	0	0	0	0	0	0.0	-0.10	0	0
138	Flushing SPE cartridge	0	10	0	0	0	2	0	0	0	0	2	0	0	0	0.0	0.0	1	0	1	0	1	0	0	0	0.0	-0.10	0	0
139	Flushing SPE cartridge	0	2	0	0	0	2	0	0	0	0	2	0	0	0	0.0	0.0	0	0	1	0	1	0	0	0	0.0	-0.10	0	0
140	Flushing product out tubing	0	30	0	0	0	2	1	0	0	0	2	0	0	0	0.0	0.0	0	1	1	0	0	0	0	0	0.0	0.00	0	0

Figure S6: A copy of the Multisyn Recipe.
